# A rare cause of sciatica: Sciatic nerve schwannoma: Report of one case with long subclinical course and misleading presentation

**DOI:** 10.1051/sicotj/2020005

**Published:** 2020-06-08

**Authors:** Renaud Maes, Pascal Ledoux, Grégoire de Brouckere

**Affiliations:** 1 Centre Hospitalier EpiCURA Baudour 7331 Baudour Belgium

**Keywords:** Schwannoma, Sciatic nerve

## Abstract

The authors report one case of schwannoma located in the sciatic nerve, just above the popliteal fossa. A sciatic localization is rare, observed in 1% of the patients. The misleading clinical presentation of this localization causes a delay in diagnosis. Magnetic Resonance Imaging (MRI) is the imaging modality of choice, but the final diagnosis is made by the histological examination of the tumor. Schwannoma should be surgically removed without division of the nerve trunk.

## Introduction

Schwannoma is a benign tumor generally observed on peripheral nerves especially in the upper limbs [1]. It is often solitary, circumscribed and encapsulated eccentrally located on spinal nerve root, or proximal nerves [2–4].

Since the identification of this tumor by Verocay in 1908, several names are used (schwannoma, neurilemoma, neurinoma, perineurial fibroblastoma, peripheral glioma, etc.) making terminology confusing, but the two most common terms utilized today is schwannoma and neurilemoma [5]. In the lower limbs, Schwannoma is often located in the posterior tibial nerve [6, 7]. A sciatic localization is rare, observed in 1% of the patients [1, 4, 8–10].

The most common manifestation of schwanomma is pain, sensory and motor dysfunction, mimicking sciatica. A painless mass can be palpated with a positive Tinel’s sign.

It often has a long subclinical course and their clinical presentation is usually misleading.

This clinical case describes a schwannoma of the sciatic nerve mimicking sciatica.

## Case report

A 50-year-old-woman presented painful path involving the left lower limb with a painful mass just above the popliteal fossa.

The initial symptoms started some months previously.

Physical examination revealed a mobile mass just above the left popliteal fossa. The mobility of the knee was normal. There was no neurologic or vascular deficit.

**Figure 1:**
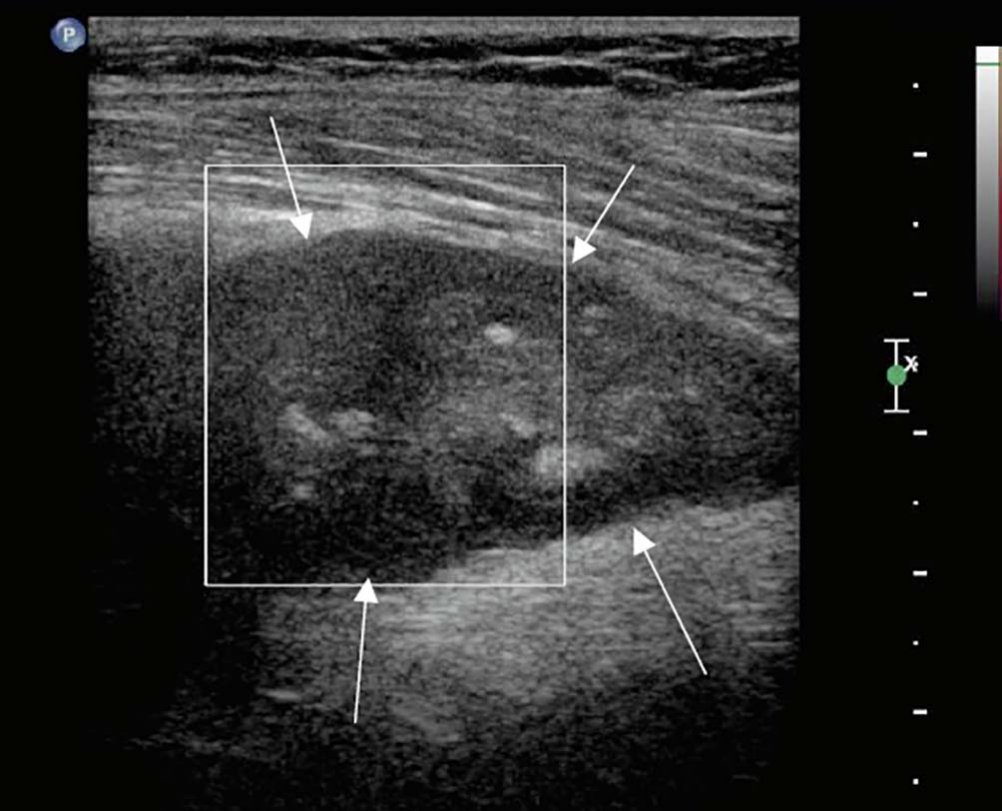
Local ultrasound shows a well-defined mass with a heterogeneous aspect with contact to the sciatic nerve (white arrows show the limit of the tumor).

Ultrasound revealed a well-defined mass with contact to the sciatic nerve in the distal posterior part of the left thigh (Figure 1).

**Figure 2:**
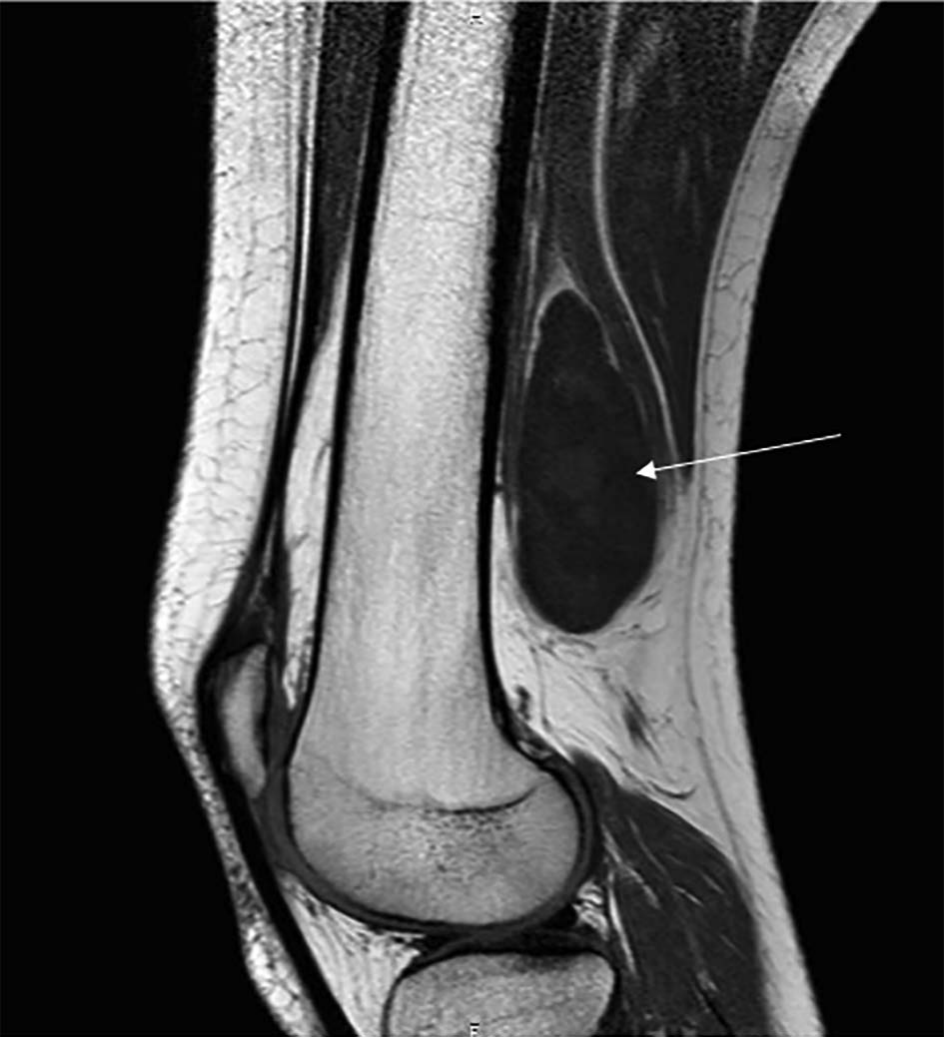
A sagittal T1-weighted MRI shows a well-defined mass, low signal intensity, inside the sciatic nerve, just above the popliteal fossa.

**Figure 3:**
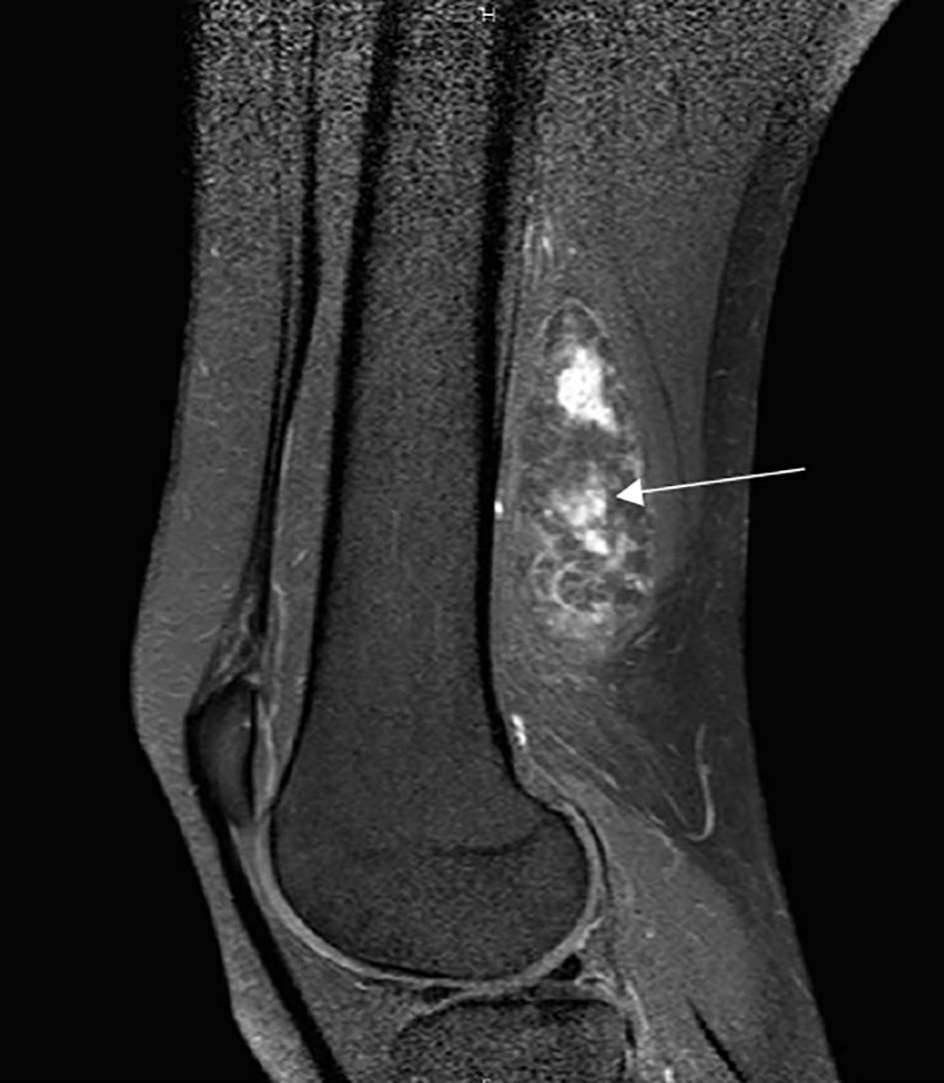
A sagittal T2-weighted MRI shows hyperintense signal in the mass with a heterogeneous aspect.

Magnetic Resonance Imaging (MRI) revealed a well-defined heterogeneous mass (7 × 4, 4 × 3, 3 cm), posterior to the sciatic nerve, just above the popliteal fossa (Figure 2). A T2-weighted MRI showed hyper intense signal in the mass (Figure 3).

Biopsy under ultrasound scan was made. Histological examination revealed a schwannoma.

February 12, 2010, the resection of the tumor was performed according to microsurgical principles.

**Figure 4:**
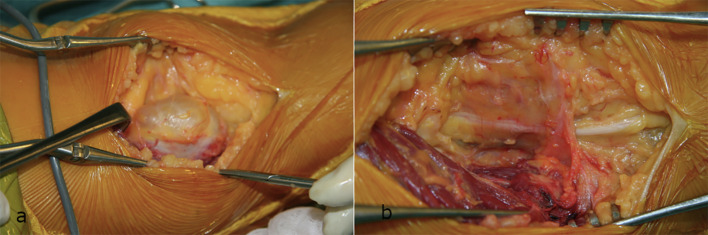
(a) Schwannoma in situ. (b) It was located just above the polpiteal fossa, originating from the sciatic nerve proximal to its bifurcation.

**Figure 5:**
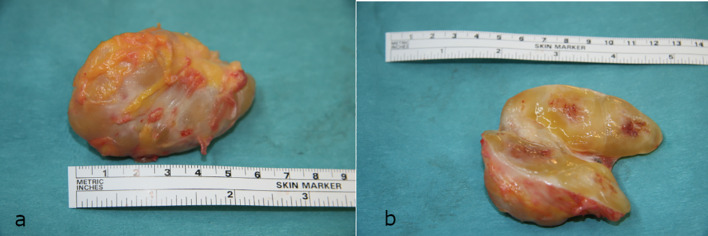
(a) Macroscopic aspect of the tumor. The size was ±6 × 4 cm. (b) Note the heterogeneous aspect inside the tumor.

The patient was placed in a prone position. A longitudinal incision was made over the left popliteal fossa. A well-delineated tumor was identified, originating from the sciatic nerve proximal to its bifurcation (Figure 4). The tumor was completely excised with preservation of the functioning fascicles (Figure 5).

**Figure 6:**
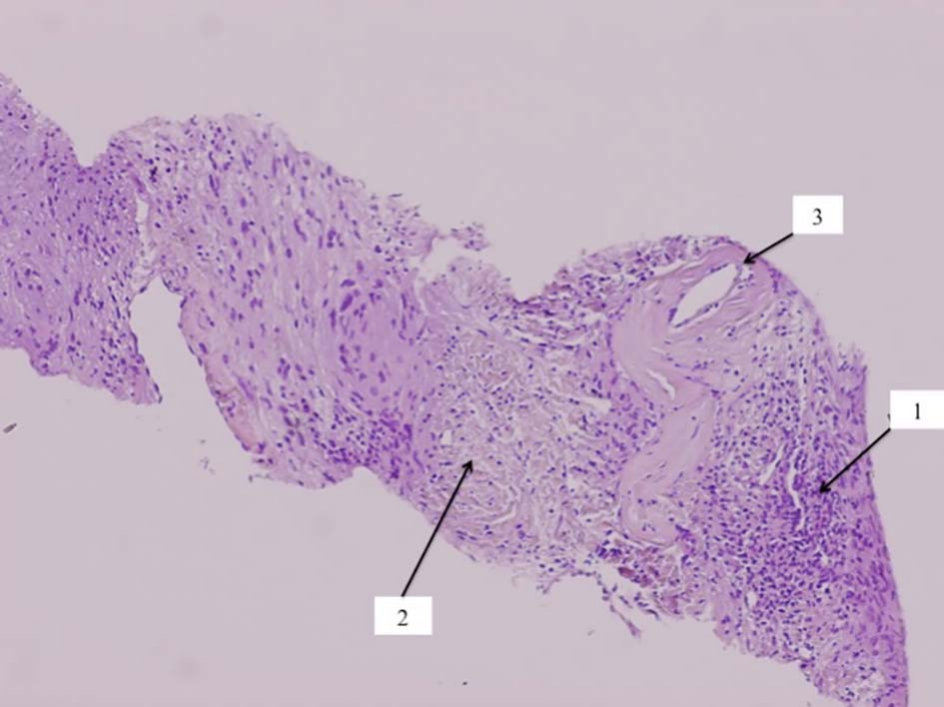
This figure clearly shows the palisading elements of the Antoni A cells (1), low cellular regions, called Antoni B areas (2) and a blood vessel with a hyalinized wall (3).

Histological analysis of the tumor showed a nodule with a capsule with high and low cellular regions called Antoni A and B areas, respectively, and a blood vessel with a hyalinized wall (Figure 6).

Postoperatively, the patient did not develop pain and motor or sensitive disturbances after a follow-up of 16 months; ultrasound scan was normal.

## Discussion

Schwannoma is rare but it is the most common benign nerve sheath tumors, especially in the upper limbs [1].

A sciatic localization is rare and it is often located in the posterior tibial nerve [6, 7].

Most schwannomas are solitary lesions. Malignant transformation occurs rarely [2–5, 11].

A schwannoma may often present as a painless, palpable mass with a positive Tinel’s sign [1, 8, 12]. The mass may be painful due to a local inflammation and can be bulky [13]. Sometimes, patients present painful numbness involving the lower limb, mimicking a sciatica. In this situation, the diagnosis of schwannoma is late and patient often seeks different medical advices. Doctors, in first, try to exclude usual causes of sciatica. Sciatica not responding to effective conservative treatment (anti-inflammatory treatment, drugs against pain, etc.), a negative Lasegue’s test, a negative lumbar scan, and a positive Tinel’s sign should suspect a peripheral nerve tumor, especially if the lesion is higher and concealed [2]. The ultrasound and MRI of the whole leg are required. These pseudo-radicular forms are difficult to diagnose [8].

In our case report, the clinical presentation was easier because the patient presented painful path involving the left lower limb with a painful mass.

The nerve conduction study and standard radiography are not contributory.

The ultrasound examination may show the mass, but the contacts with the nerve are not easy to study [14]. MRI identified the tumor and its contacts with the nerve. It showed a well-circumscribed and eccentrally mass located on the nerve [2, 9, 10].

The final diagnosis is possible by histological analysis [9].

Enzinger and Weis described the several microscopic feature characteristic of a schwannoma, found in our case. They are: (1) high and low cellular regions called Antoni A and B areas, respectively; (2) in the Antoni A area, there may be foci of palisaded nuclei called Verocay bodies; (3) relatively thick capsule [2, 3, 15].

Surgical treatment is the treatment of choice [2, 4, 16, 17]. Schwannoma must be excised without division of the nerve trunk because it permits expectation of normal function without sensory or motor disturbances.

In the literature, the incidence of recurrence is low [16] and it may due to a consequence of incomplete resection [5, 11].

In conclusion, the persistence of sciatica despite a negative routine workup should indicate the possibility of peripheral nerve neoplasms. MRI is the radiological examination of choice, but the final diagnosis is made by the histological examination of the tumor.

The treatment of choice of schwannoma remains the surgical resection without division of the nerve trunk.

The incidence of recurrence is low and may due to an incomplete resection.

## Conflict of interest

The authors declare that they have no conflict of interest.
